# A Rare Localization of Actinomycosis Mimicking Ulcerative Malignancy

**DOI:** 10.1155/2013/323210

**Published:** 2013-03-04

**Authors:** Luca Volpi, Fabio Ferreli, Maurizio Bignami, Andrea Pistochini, Francesco Meloni, Apostolos Karligkiotis, Paolo Castelnuovo

**Affiliations:** ^1^Department of Otorhinolaryngology, University of Insubria, Varese, Italy; ^2^Department of Otorhinolaryngology, University of Sassari, Sassari, Italy

## Abstract

Actinomycosis is a chronic, suppurative, and granulomatous process caused by Actinomycetes, saprophytic bacteria normally residing in the oral cavity. It can involve any organ, but the cervicofacial disease is the most frequent. Pharyngolayngeal involvement is rare and usually occurs secondary to the oral or cervical disease. There are few cases of primary pharyngolaringeal actinomycosis described in the literature. A rare case of pharyngeal actinomycosis mimicking an ulcerative malignancy in a 63-year-old man is reported. The patient was treated successfully with long-term antibiotic therapy. The clinical and pathological features and the aspects of diagnosis and treatment of cervicofacial actinomycosis are discussed.

## 1. Introduction 

Actinomycosis is an uncommon bacterial infection characterized by a chronic, suppurative, and granulomatous process due to Actinomycetes. They are usually saprophytic bacteria of the oral cavity and the digest tract, but sometimes can lead to local and diffuse infections. The infection is caused by a mixture of microbes with a predominance of the *Actinomyces israelii*, a gram-positive anaerobic bacillus. Five species of Actinomycetes have been identified: *israelii, bovis, naeslundii, viscous,* and *odontolyticus*. All these species are normal flora of the oral cavity with exception of *bovis* [1]. 

In 1938 Cope first classified actinomycosis into 3 different forms: cervicofacial, pulmonothoracic, and abdominopelvic, respectively, 50%, 30%, and 20% of cases [2]. The predisposing factors are represented by debilitating conditions such as malignancy, diabetes, and immunosuppression [3]. Cervicofacial actinomycosis is also more frequent in people with poor oral hygiene and oral mucosal trauma. The fifth decade of life is the most affected, and there is a little male prevalence. Actinomycosis located at the cervicofacial district classically presents a slowly growing, firm, painless, and possibly suppurating submandibular mass, but it can also present a rapidly progressive, painful, and fluctuant infection anywhere in the neck or face associated with fever and leukocytosis. Racial predisposition or geographic factors are unknown.

 Actinomycosis is an insidious disease, and its propensity to mimic different pathologies, such as tuberculosis or carcinoma, is well known. CT and MR are aspecific for diagnosis, but they can help in defining the localization and the extension of the lesion [4]. The certain diagnosis is based on cytology (FNAC) and/or biopsy [5]. We report a rare case of pharyngeal actinomycosis mimicking an ulcerative malignancy.

## 2. Case Report 

**Figure 1:**
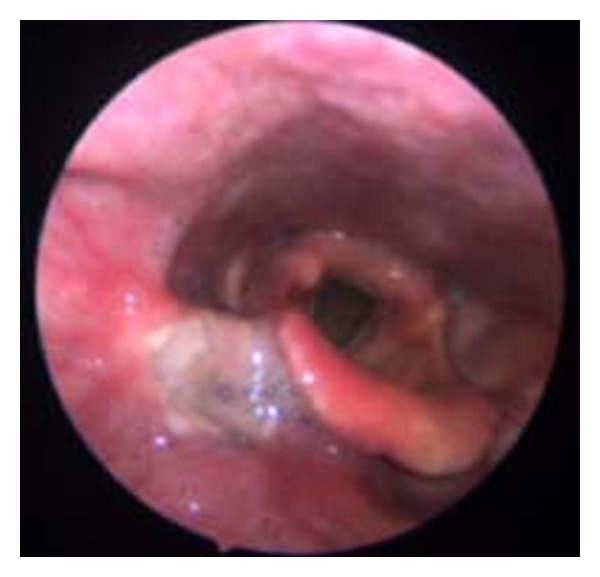
Endoscopic view evidences an ulcerative lesion on the right pharyngoepiglottic ligament, homolateral vallecula, and the right pyriform sinus.

**Figure 2:**
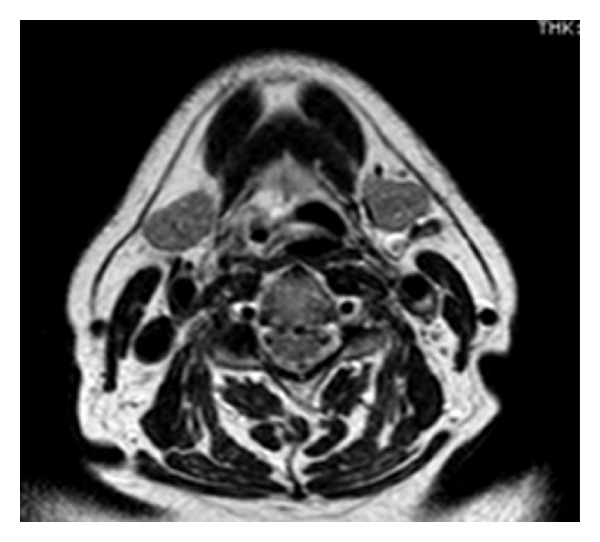
MR image shows an irregular area of tissue thickening with moderate contrast enhancement on the right pharyngo-epiglottic ligament and omolateral pyriform sinus.

**Figure 3:**
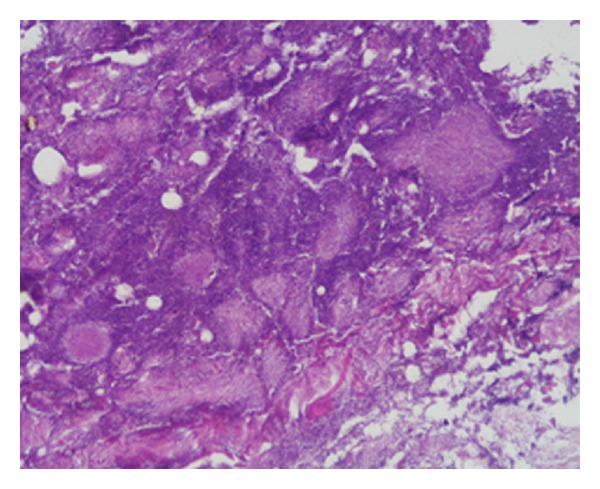
Hematoxylin-eosin stain 20x. Bacterial colonies situated inside the neutrophilic collections create characteristic structures called “sulfur granules.”

**Figure 4:**
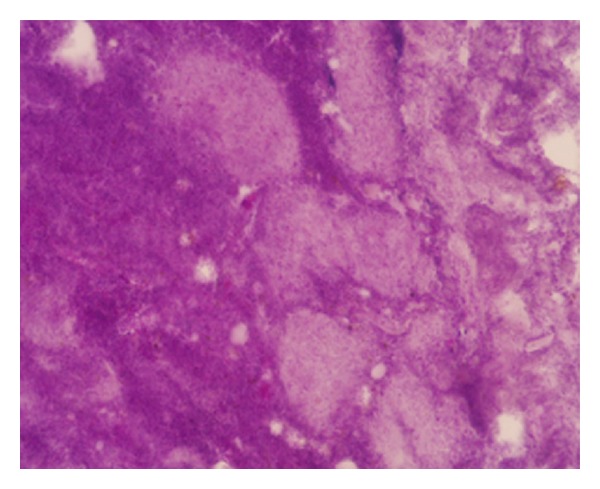
PAS stain 40x. Internal bacteria stained with the PAS procedure.

A 63-year-old man, non smoker, affected by diabetes mellitus and ischemic heart disease, complained of episodes of hemoptoe. He did not refer to dyspnoea or fever, and no previous surgery in the head and neck region was reported. Our examination of the oral cavity was normal, and there were no dental pathologies. Fiber optic laryngoscopic evaluation revealed an ulcerative lesion on the right pharyngoepiglottic ligament, homolateral vallecula, and right pyriform sinus (Figure 1).Diagnostic suggestion appeared to be a neoplastic process. Clinical evaluation of the neck did not show lymphadenopathy or skin alteration. Routine blood test evidenced only mild anemia (12,1 mg/dL), and chest X-ray was normal. Neck ultrasound was negative for cervical lymphadenopathies. MR confirmed the presence of an irregular tissue thickening with moderate contrast enhancement on the right pharyngoepiglottic ligament and homolateral pyriform sinus, but the radiologic findings were not specific (Figure 2). In microlaryngoscopy under general anaesthesia, a biopsy of the lesion was performed for histological diagnosis. Histologically a necrotizing granulomatous reaction with central aggregates of neutrophils, forming microabscesses, was observed. Some bacterial colonies were situated inside the neutrophilic collections and they formed characteristic structures that have been called “sulfur granules” (Figure 3). The internal bacteria were also stained with the PAS procedure (Figure 4).

After histologic diagnosis of actinomycosis, oral antibiotic therapy was administered with a regimen of amoxicillin 500 mg 3 times a day. It was planned to continue this for 3 months. MR after 1 month revealed a reduction of the lesion. Fiber optic laryngoscopic evaluation and MR performed after 3 months showed no evidence of disease.

## 3. Discussion

*Actinomyces* are normal inhabitants of the human oral cavity that cannot penetrate healthy tissue, so mucosal breakdown is a predisposing factor for infection. It is very important to investigate if the patient has any risk factors to suspect actinomicosis such as poor oral hygiene, malignancies, diabetes, and immunosuppression [6, 7]. In the present case, the patient denied any clinical history of oromaxillofacial trauma, but he was affected by diabetes, that is correlatable with a debilitating condition. The fifth decade of life is the most affected, and there is a little male prevalence. Racial predisposition or geographic factors are unknown [8]. Actinomycosis of the cervicofacial district usually occurs as a firm mass in submandibular region associated with surrounding hardening or erythema, with slowly growing, painless, and possibly suppurating mass, but it can also present a rapidly progressive fluctuant mass, painful, associated with fever and leukocytosis [9]. Clinically, the absence of lymphadenopathy in the presence of marked induration may differentiate actinomycosis from other diseases such as tubercclosis, syphilis, and sarcoma. In our case the neck ultrasound and the MR did not show cervical lymphadenopathy. Actinomycosis is an insidious disease and its propensity to mimic different pathologies, such as tuberculosis or carcinoma, is well known. CT and MR are aspecific for diagnosis, and they can help in defining site and extension of the lesion [6]. A recent radiologic study evidences the importance of imaging to show the extension of a pharyngeal actinomycosis to the adjacent neck space crossing fascial plane [5]. The invasion of the fascial plane can be related to the bacterial infection spreading without respect for anatomical structures or lymphatic drainage. This infiltrative nature can be correlated with proteolytic enzymes released by Actinomycetes. Typically most lesions appear as not well-defined, infiltrative, soft-tissue masses with an inflammatory reaction.

In our case the MR showed an irregular and undefined tissue thickening with moderate contrast enhancement on the right pharyngoepiglottic ligament and homolateral pyriform sinus, but these radiologic findings were not specific. 

The certain diagnosis is based on cytology (FNAC) and/or biopsy [7]. In our case the site of the lesion needed a biopsy in microlaryngoscopy under general anaesthesia.

The histological examination revealed characteristic sulfur granules on a hematoxylin-eosin-stained section. These are rounded or elongated, deep purple aggregates composed of filamentous organisms. The sulfur granules often have eosinophilic club-shaped ends and are often encrusted with protein in the Splendore-Hoeppli phenomenon.

Pharyngeal actinomycosis is a rare localization. Actinomycosis shows a wide variety of symptoms and a characteristic ability to mimic many other diseases. Because of its peculiarity it can be considered a “great pretender” [8]. Only 10% of *Actinomyces* infections are correctly diagnosed on initial presentation [9]. In the past, surgery has been used both to diagnose and to treat this pathology with its removal. Nowadays with the advent of FNAC, the diagnosis has become easier and less invasive. The biopsy can be performed to obtain the correct diagnosis when multiple FNACs are not diagnostic or the site of the lesion is impossible to reach, as in our case. Moreover the surgical treatment is necessary when complications associated with actinomycosis, such as an abscess to be drained, occur. The main therapeutic treatment is administration of antibiotics, and penicillin is the drug of choice. Erythromycin and tetracycline can be used in patients allergic to penicillin [10]. The antibiotic therapy must be administered in high dosage over a prolonged period because of the tendency of the disease to recur.
